# Prognostic significance of immunohistochemically detected breast cancer node metastases in 218 patients

**DOI:** 10.1038/sj.bjc.6600420

**Published:** 2002-07-15

**Authors:** I de Mascarel, G MacGrogan, V Picot, S Mathoulin-Pelissier

**Affiliations:** Department of Pathology, Institut Bergonié, Comprehensive Cancer Center, 180 rue de Saint-Genès, 33076 Bordeaux Cedex, France

**Keywords:** breast axillary node metastases, immunohistochemical stainings, prognostic significance, follow-up

## Abstract

Axillary lymph node metastases detected by immunohistochemistry in standard node-negative patients with breast carcinomas (13 out of 129 infiltrating ductal carcinomas and 37 out of 89 infiltrating lobular carcinomas) do not have any prognostic significance in patients followed up for a long time (respectively 24 and 18 years). Moreover, their pejorative significance in the literature is debatable since the groups and events taken into account are heterogeneous.

*British Journal of Cancer* (2002) **87**, 70–74. doi:10.1038/sj.bjc.6600420 www.bjcancer.com

© 2002 Cancer Research UK

## 

Previous studies on the prognostic significance of axillary node metastases detected by immunohistochemical stainings (IHM) in invasive breast cancer have focused on a variable number of cases with different histological sampling techniques and statistical methods. The prognostic significance of such metastases is still debated and their clinical management is controversial. In our two previously published groups (Trojani *et al*, 1987a,b) of patients (grouped together in the present study under the name ‘study 1’–1987), nodal metastases detected by immunohistochemistry were associated with shorter metastasis-free probability (MFP) and overall survival probability (OSP) in the infiltrating ductal carcinoma node-negative group of patients (IDC, median follow-up: 10 years, Trojani *et al*, 1987a) but not in the infiltrating lobular carcinoma node-negative group of patients (ILC, median follow-up: 6.5 years, Trojani *et al*, 1987b). In the same two groups of patients with a longer follow-up (median follow-up: 15.6 years in the IDC group and 9.3 years in the ILC group, ‘study 2’–1992, de Mascarel *et al*, 1992), these IHM were still associated in the IDC group with a shorter MFP, but survival was not different between patients with or without metastases. In the ILC group there was still no difference in MFP and OSP between patients with or without metastases.

The aim of the present study (‘study 3’–2001) was to use longer follow-up to assess the prognostic significance of metastases detected by immunohistochemical stainings in these two IDC and ILC groups of patients with node-negative breast carcinomas.

## MATERIALS AND METHODS

### Patients

From 1965 to 1984, 2768 patients with distant metastasis-free breast cancer underwent surgery at Institut Bergonié. They were prospectively included in our clinical, histological and biological database and followed up at our institution. In 1987 129 node-negative patients were selected with infiltrating ductal carcinomas (IDC) operated on between 1965 and 1977 (Trojani *et al*, 1987a) and 89 node-negative patients with infiltrating lobular carcinoma (ILC) operated on between 1965 and 1984 (Trojani *et al*, 1987b). All slides of tumours and lymph nodes were reviewed by a senior pathologist (IM) and the distribution of clinical and pathological criteria are summarised in Table 1

**Table 1:**
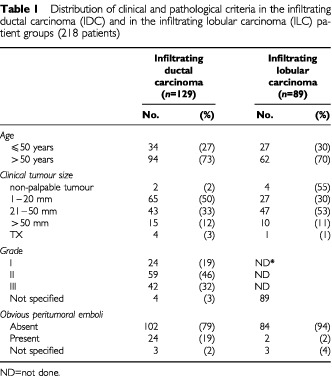
Distribution of clinical and pathological criteria in the infiltrating ductal carcinoma (IDC) and in the infiltrating lobular carcinoma (ILC) patient groups (218 patients)

. All the patients were treated by Patey type mastectomy and axillary node dissection (only five patients without IHM in the IDC group and one in the ILC group received a brief course of chemotherapy). In the IDC and ILC groups, respectively 24 and 30 patients received radiotherapy. Among the 129 patients with IDC (median follow-up: 24 years), 26 had distant metastases (20%) and 67 died (52%). Among the 89 patients with ILC (median follow-up: 18 years), 16 had distant metastases (18%) and 37 died (41.5%).

### Macroscopic lymph node processing: macroscopic serial sectioning

**Figure 1:**
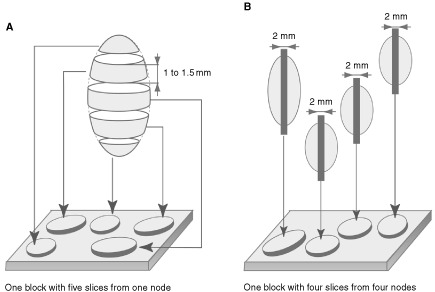
Macroscopic lymph node processing. (**A**) Macroscopic serial sectioning. (**B**) Standard sectioning.

The mean number of lymph nodes analysed in each case was 14 (range 2–29). Since 1965 all axillary lymph nodes have been examined at our institute by macroscopic serial sectioning. After fixation in Bouin-Holland, each node is macroscopically cut entirely into 1–1.5 mm thick slices perpendicular to the long axis (one to nine slices, mean: four). All slices of one node are placed together in as many numbered cassettes as necessary and paraffin-embedded. The number of cassettes (paraffin blocks) required to analyse each entire node ranged from one (90% of the cases) to three. Each block is examined on one haematoxylin-eosin-safran (HES) stained slide. Thus, in 90% of the cases all the slices of one node were situated on one HES slide (Figure 1A

).

### Immunohistochemical stainings

Immunostaining was performed on the original diagnostic HES-stained slides of the axillary nodes. These were the same sections in which metastases were considered to be negative by routine HES examination. They were successively destained and restained by a three-stage immunoperoxidase procedure with a cocktail of five monoclonal antibodies against epithelial cell antigens (Trojani *et al*, 1987a,b). IHM were found in 37 ILC (41%) and in 13 IDC (10%). They were detected in only one lymph-node per axillary node dissection in the IDC group and in one (26%), two (6%), three (6%) or four (3%) lymph nodes per dissection in the ILC group. In all the cases IHM were unequivocal but morphologically different according to the histological type. In IDC, they corresponded to small tumour cell clusters in the subcapsular sinuses ranging from 0.01 to 0.2 mm in size, whereas in ILC, they corresponded to a variable number of isolated tumour cells with an irregular distribution, sometimes throughout the entire node sections. These isolated cells were neither counted nor measured.

### Statistical analysis

Metastasis-free probability (MFP) and overall survival probability (OSP) were calculated from the date of surgery to the occurrence of distant metastasis or to death. Life tables were calculated according to the Kaplan–Meier method. In the IDC and ILC groups, we compared MFP and OSP between the patients with and without IHM using the log-rank test (software SPSS 9.01, SPSS Inc 1989–1999).

## RESULTS

The distribution of distant metastases and deaths in relation to the presence or the absence of node metastases detected by immunohistochemistry in the two groups is summarised in Table 2

**Table 2:**
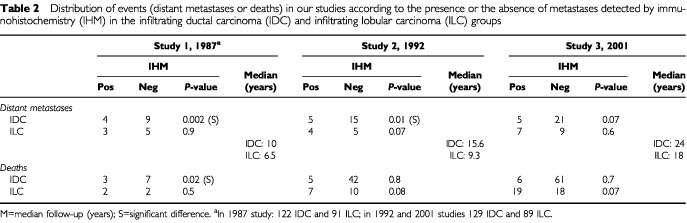
Distribution of events (distant metastases or deaths) in our studies according to the presence or the absence of metastases detected by immunohistochemistry (IHM) in the infiltrating ductal carcinoma (IDC) and infiltrating lobular carcinoma (ILC) groups

**Figure 2:**
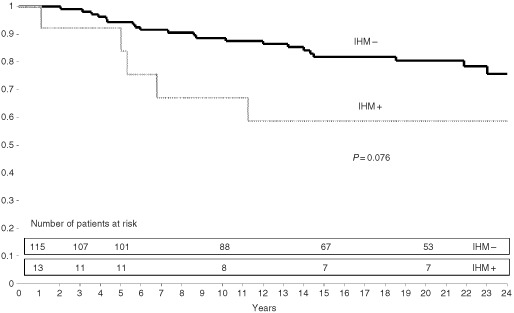
Metastasis-free survival according to presence or absence of node metastases detected by immunohistochemistry (IHM) in IDC group.

. In neither group were there any significant differences in MFP (Figures 2

and 3

**Figure 3:**
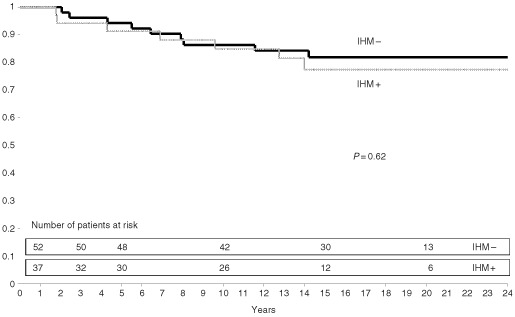
Metastasis-free survival according to presence or absence of node metastases detected by immunohistochemistry (IHM) in ILC group.

) or OSP between patients with and without IHM.

## DISCUSSION

### Analysis of our study

The relatively small number of patients and events requires caution in the interpretation of these results. Nevertheless, our ILC group is the largest published to date. In 1987, events taken into account to calculate survival probabilities were different from now and included distant metastases and loco-regional recurrences for recurrence-free probability and only deaths from cancer for survival. However, the results are the same when taking into account the same events as those in 1987. Furthermore, as regards patients who received radiotherapy *vs* those who did not, it has been proved that radiotherapy decreases locoregional recurrences but has no influence on MFP or OSP in node–negative patients (Rutqvist *et al*, 1993). Lastly, although the serial macroscopic sectioning method is now recommended (Fitzgibbons *et al*, 2000), our study is the only series to date with serial macroscopic sectioning and with such a long follow-up. In our series, serial macroscopic sectioning and IH stainings on the original sections may explain the small size of IHM, all of which were occult metastases. They could be termed micrometastases in the IDC group because they were much smaller than 2 mm in size. On the other hand, the use of such a term is debatable in the ILC group since they correspond to a variable number of isolated tumour cells which were irregularly distributed, sometimes throughout the entire lymph node sections. Finally, our results underline the importance of length of follow-up in assessing the prognostic significance of metastases detected by immunohistochemistry, since the difference in MFP between patients with and without IHM was no longer statistically significant in the IDC group. Whatever the cases, differences in MFP at 10 years may still be clinically relevant even if no differences are subsequently apparent, although they do not justify using such a single criterion to initiate chemotherapy. On the contrary, in ILC the difference between true node-negative and IHM was not significant at any time point. Thus, these differences in frequency, histological pattern and variability of prognostic significance according to histological type suggest that a difference in nature might exist between IHM in IDC and IHM in ILC.

### Analysis of other studies

Some authors have attempted to summarise studies on axillary micrometastases (Dowlatshahi *et al*, 1997), but the complexity and heterogeneity of the methodologies used have made the task difficult. We analysed the results of the 11 published series regarding the prognostic significance of metastases detected by immunohistochemistry (Byrne *et al*, 1987, 1992; Trojani *et al*, 1987a,b; Sedmak *et al*, 1989; Chen *et al*, 1991; Galea *et al*, 1991; Noël *et al*, 1991; de Mascarel *et al*, 1992; Elson *et al*, 1993; Hainsworth *et al*, 1993; Nasser *et al*, 1993; McGuckin *et al*, 1996; Cote *et al*, 1999) by comparing size and type of populations, histological tumour types, lymph node processing, immunohistochemical stainings and statistical analyses (Table 3

**Table 3:**
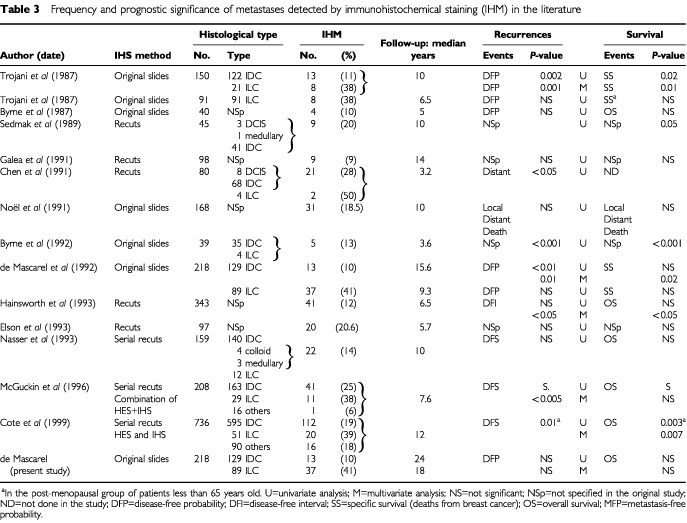
Frequency and prognostic significance of metastases detected by immunohistochemical staining (IHM) in the literature

). Only one series was prospective (Cote *et al*, 1999). Contrary to our study, a standard macroscopic technique was used in all the other studies, i.e. each lymph node was examined on one 2–3 mm thick slice transected in the major axis (Figure 1B). The mean number of lymph nodes per axillary node dissection was variable, and immunohistochemical stainings were performed either on original destained slides (Byrne *et al*, 1987; Trojani *et al*, 1987a,b; Noël *et al*, 1991; Byrne *et al*, 1992) or on slides from recuts of each block (Sedmak *et al*, 1989; Chen *et al*, 1991; Galea *et al*, 1991; Elson *et al*, 1993; Hainsworth *et al*, 1993; Nasser *et al*, 1993; McGuckin *et al*, 1996; Cote *et al*, 1999). The latter approach cannot distinguish between metastases that would be identifiable at deeper levels without immunohistochemistry and cases that are detectable only with immunohistochemical staining. The percentages of IHM according to the histological tumour type have been studied in only four reports (Trojani *et al*, 1987a,b; Byrne *et al*, 1992; McGuckin *et al*, 1996; Cote *et al*, 1999). When specified, definitions of events taken into account to calculate survival probabilities were heterogeneous and median follow-up was variable. All these differences in methodologies explain why neither the detection rates of these IHM nor their prognostic significance are comparable. IHM were associated with poorer prognosis in five studies (Sedmak *et al*, 1989; Byrne *et al*, 1992; Hainsworth *et al*, 1993; McGuckin *et al*, 1996; Cote *et al*, 1999). This prognostic significance is debatable due to the small number of patients (Sedmak *et al*, 1989; Byrne *et al*, 1992), the short median follow-up (Hainsworth *et al*, 1993), and to the fact that IHM were detected not only by immunohistochemistry but by a combination of morphological analysis on haematoxylin-stained slides and immunohistochemistry (McGuckin *et al*, 1996). In the study by Cote *et al* (1999), IHM were associated with a shorter survival by univariate analysis in the under 65-year-old post-menopausal group of patients corresponding to 7% (53 out of 736) of the patients in their series. Multivariate analysis was performed on groups of patients stratified according to oestrogen receptor (ER) status (progesterone receptor status not specified), so the prognostic significance (value of risk) of IHM *vs* ER status is debatable. Furthermore, the relative importance by multivariate analysis of IHM *vs* tumour size, grade and vascular invasion was not specified. Lastly, perioperative chemotherapy was not effective in the group of patients in whom IHM were found.

## CONCLUSION

On the whole, the pejorative significance of breast axillary node metastases detected by immmunohistochemistry is debatable. It has to be proved before using immunohistochemical stainings as a standard in the sentinel lymph node technique. In conclusion: (1) our results emphasise the importance of length of follow-up in assessing the significance of metastases detected by immunohistochemistry; (2) in the literature there is no firm evidence underlining the prognostic significance of such metastases, and (3) more prospective and concordant studies are necessary to confirm or not the prognostic significance of metastases detected by immunohistochemistry. Therefore, a standard methodology is required in the pathological assessment of axillary lymph nodes.
